# Impact of Different Etching Strategies on Margin Integrity of Conservative Composite Restorations in Demineralized Enamel

**DOI:** 10.3390/ma13204500

**Published:** 2020-10-11

**Authors:** Mohamed El Gedaily, Thomas Attin, Daniel B. Wiedemeier, Tobias T. Tauböck

**Affiliations:** 1 Department of General, Special Care, and Geriatric Dentistry, Center for Dental Medicine, University of Zurich, 8032 Zurich, Switzerland; mohamed.elgedaily@zzm.uzh.ch; 2 Department of Conservative and Preventive Dentistry, Center for Dental Medicine, University of Zurich, 8032 Zurich, Switzerland; thomas.attin@zzm.uzh.ch; 3 Statistical Services, Center for Dental Medicine, University of Zurich, 8032 Zurich, Switzerland; daniel.wiedemeier@zzm.uzh.ch

**Keywords:** demineralized enamel, marginal adaptation, phosphoric acid etching, resin composite, self-etch adhesives

## Abstract

Good margin integrity with a tight seal of the adhesive interface is considered one of the key factors for the clinical success of composite restorations. This study investigated the effect of enamel etching with phosphoric acid on the margin integrity of self-etch bonded composite restorations in demineralized enamel. Crowns of bovine incisors were assigned into 14 groups (n = 10 per group) of which ten groups (groups 1–5 and 8–12) were demineralized (21 days, acid buffer, pH 4.95) to create artificial carious lesions. Standardized Class V cavities were prepared in all specimens. Demineralized groups were either etched with phosphoric acid for 10, 30, 60, or 120 s (groups 2–5 and 9–12), or no etching was performed (groups 1 and 8). The non-demineralized (sound) groups were etched for 10 s (groups 7 and 14) or remained non-etched (groups 6 and 13). Resin composite restorations were then placed using either a one-step (iBond Self Etch, groups 1–7) or two-step self-etch adhesive (Clearfil SE Bond, groups 8–14). Margin integrity of the restorations was assessed after thermocycling (5000×, 5–55 °C) using scanning electron microscopy, and the percentage of continuous margins (%CM) was statistically analyzed (α = 0.05). Phosphoric acid etching significantly increased %CM in both demineralized and sound enamel. For iBond Self Etch, a significant increase in %CM in demineralized enamel was observed with increased etching times. All etched groups treated with Clearfil SE Bond and those etched for 60 or 120 s and treated with iBond Self Etch showed similar %CM in demineralized enamel as in etched sound enamel, and significantly higher %CM than in non-etched sound enamel. In conclusion, enamel etching with phosphoric acid improves margin integrity of composite restorations in demineralized enamel when bonded with the examined adhesives.

## 1. Introduction

Resin composites are widely used and recommended as restorative materials because of their good physicomechanical properties and the possibility to adhesively bond them to dental hard tissues [1,2,3,4,5]. These characteristics allow for minimally invasive, defect-oriented treatments without the need of macro-mechanical retention [6,7]. In daily routine, the process of cavity preparation for a composite restoration often includes excavation of softened and infected dentin as well as the complete removal of demineralized enamel areas [8]. Omitting to extend restoration margins to sound enamel may save large areas of dental hard tissue and prevent disproportionate tooth substance loss. It would therefore be important to know whether restoration margins can be placed in demineralized enamel and still attain sufficient sealing ability. Given that interfacial gap formation may lead to subsequent problems such as margin discoloration and secondary caries [9,10], reliable marginal adaptation of composite restorations is essential to achieve clinical success [11].

Other than etch-and-rinse systems, self-etch adhesives allow to bond composite materials to teeth without preceding phosphoric acid etching, and thus simplify handling and reduce technique sensitivity of adhesive treatments [12]. While two-step self-etch adhesives are used by applying a self-etching primer followed by an adhesive resin, one-step self-etch adhesives contain all components required for etching, priming, and bonding in one solution. One- and two-step self-etch adhesives have been shown to attain similar enamel bond strengths [13]. A recent study [14] revealed that the margin integrity of composite restorations bonded with a self-etch adhesive was significantly lower in demineralized enamel than in sound enamel, while no decline in margin integrity was observed in demineralized vs. sound enamel when etch-and-rinse adhesives were used. Other studies proved that margin integrity of composite restorations increased after selective etching of sound enamel before application of a self-etch adhesive [15,16]. Thus, the question arises, whether margin integrity of composite restorations in demineralized enamel may also benefit from pre-etching with phosphoric acid.

Based on these considerations, the aim of this in vitro study was therefore to investigate the effect of selective enamel etching with phosphoric acid, applied for different etching times, on the margin integrity of composite restorations in demineralized enamel when using a one-step and two-step self-etch adhesive, respectively. The null hypothesis tested was that selective etching of demineralized enamel would have no effect on the margin integrity of composite restorations bonded with self-etch adhesives.

## 2. Materials and Methods

### 2.1. Specimen Preparation and Demineralization

**Figure 1:**
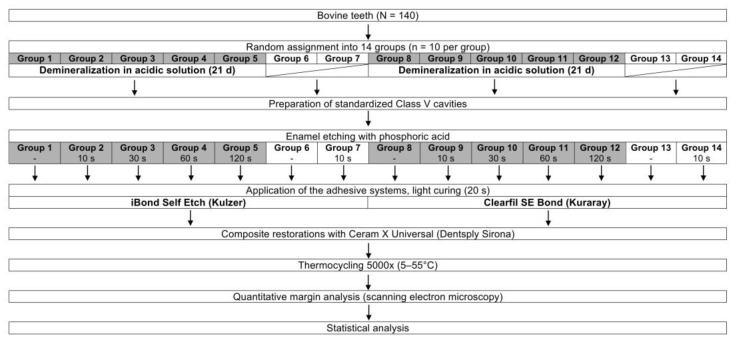
Experimental design.

One hundred and forty crowns of freshly extracted permanent bovine incisors, stored in tap water until use [14,17], were chosen for this in vitro study. The pulp cavum of each tooth was filled with plasticine (Pelikan, Hanover, Germany) and sealed with nail polish to prevent internal demineralization. The teeth were polished (Sof-Lex Pop-on superfine, 3M ESPE, St. Paul, MN, USA) to remove the cementum layer from the enamel [14,17], and randomly allocated into 14 groups (n = 10 per group). The experimental design is illustrated in Figure 1.

Specimens of groups 1–5 and 8–12 were demineralized according to a previously established protocol to create artificial carious lesions [14,17]. For this purpose, the teeth were put for 21 days in an acidic solution (pH 4.95) at 37 °C containing 3 mM CaCl_2_·2H_2_O, 3 mM KH_2_PO_4_, 6 µM MHDP, KOH to adjust the pH to 4.95, 50 mM CH_3_COOH, and distilled water [18]. The acidic solution was changed on a daily basis to keep the pH constant. Afterwards, standardized buccal cavities (diameter: 3 mm, depth: 2 mm, bevel: 1 mm) with circumferential enamel margins were prepared in all teeth using spherical diamond burs (D126, Garant, Munich, Germany).

### 2.2. Adhesive Pretreatment

Selective enamel etching with 37% phosphoric acid (Ultradent, South Jordan, UT, USA) was performed using different etching times. Groups 2, 7, 9, and 14 were etched for 10 s, groups 3 and 10 for 30 s, groups 4 and 11 for 60 s, and groups 5 and 12 for 120 s, before rinsing the cavities with water for 30 s and drying gently with air. Groups 1, 6, 8, and 13 remained unetched. Thereafter, groups 1–7 were treated with the one-step self-etch adhesive iBond Self Etch (Kulzer, Hanau, Germany), while groups 8–14 were treated with the two-step self-etch adhesive Clearfil SE Bond (Kuraray, Osaka, Japan) according to manufacturers’ instructions. The composition of the two self-etch adhesives is detailed in Table 1. iBond Self Etch (pH = 1.6–1.8) was applied to the cavities, agitated for 20 s with an application tip, carefully air-dried, and light cured for 20 s. The specimens of the Clearfil SE Bond groups were treated by first applying Clearfil SE Bond Primer (pH = 2.0) to the cavities with an application tip, leaving it in place for 20 s and gently air-drying it. Then, the Clearfil SE Bond bonding agent was applied, air-dried, and light cured for 20 s. Light curing was conducted with an LED curing unit (Bluephase G2, Ivoclar Vivadent, Schaan, Liechtenstein) at an irradiance of ≥1100 mW/cm^2^, which was checked regularly with a calibrated power meter (FieldMaxII-TO, Coherent, Santa Clara, CA, USA).

### 2.3. Restoration and Thermocycling

The cavities were restored in one increment with a nano-hybrid composite (Ceram X Universal, Dentsply Sirona, Konstanz, Germany) and light cured for 20 s. Excess of composite was removed with surgical scalpel blades (No. 12D, Gebr. Martin, Tuttlingen, Germany), and the restorations were polished under a microscope at 25× magnification (Stemi 2000, Zeiss, Oberkochen, Germany) using silicon instruments (Brownie Mini-Points and Greenie Mini-Points, Shofu Dental Corporation, San Marcos, CA, USA) and polishing brushes (Occlubrush, Kerr, Orange, CA, USA).

The specimens were then thermocycled by dipping them 5000 times alternately in water baths with temperatures of 5 and 55 °C, with a dwell time of 20 s in each bath and a transfer time of 10 s [14,17].

### 2.4. Assessment of Margin Integrity

After thermocycling, replicas of the specimens were made by first producing negative copies with an A-silicone (President Light Body, Coltène, Altstätten, Switzerland), which were then poured with epoxy resin (Epoxyharz L, R&G Faserverbundwerkstoffe, Waldenbuch, Germany). The replicas were coated with gold using a sputtering device (Sputter SCD 030, Balzers Union, Balzers, Liechtenstein), and a quantitative margin analysis was performed using scanning electron microscopy at 20 kV and 200× magnification (Vega TS5136XM, Tescan, Brno, Czech Republic). Margin qualities of the restorations were categorized as “continuous”, “non-continuous”, or “not judgeable” using a customized self-programmed application based on 4D (4D SAS, Le Pecq, France) according to previous research [14,19,20]. The margin integrity was then expressed as a percentage of continuous margins in relation to the total length of judgeable continuous and non-continuous margins [17,21], and statistically analyzed.

### 2.5. Statistical Analysis

The data were tentatively fit into a two-way ANOVA; however, assumptions about homogeneity and normality of residuals were violated. Thus, the experimental groups within the two adhesives were analyzed separately using the Kruskal–Wallis test and post-hoc Conover tests. *p*-values were adjusted for multiple testing according to Holm. Moreover, the analogue experimental groups from both adhesives were compared pairwise using the Wilcoxon rank sum test, while *p*-values were again adjusted for multiple testing according to Holm. The entire data analysis was conducted using the open-source statistical environment R [22], including the package PMCMR [23]. The level of significance was set at α = 0.05.

## 3. Results

**Figure 2:**
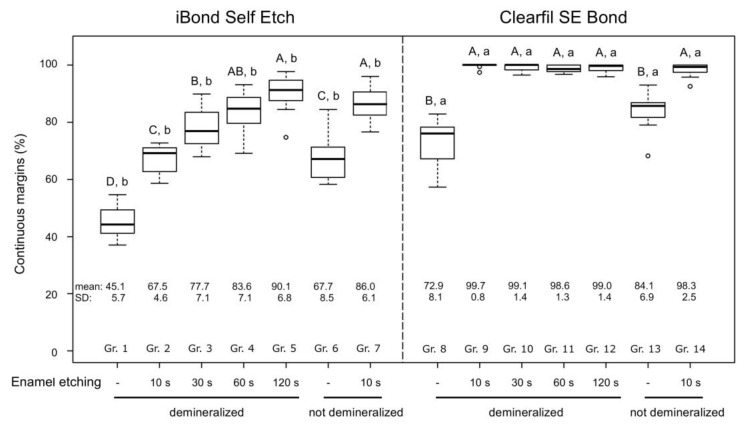
Continuous margins (%) of the composite restorations in demineralized and not demineralized (sound) enamel after application of the tested adhesive systems (iBond Self Etch, Clearfil SE Bond) with different enamel etching times. The thick lines within the boxplots represent the medians, whereas the upper and lower margins of the boxes are equivalent to the 75% and 25% quartiles, respectively. The whiskers illustrate 1.5 × interquartile range (IQR), or maxima and minima of the distribution if below 1.5 × IQR. Circles represent the outliners. Significant differences between groups within each adhesive system are indicated with different capital letters (post-hoc Conover tests, *p* < 0.05), while significant differences between analogue experimental groups of the two adhesives are indicated with different small letters (Wilcoxon rank sum test, *p* < 0.05). SD: standard deviation.

The percentages of continuous margins (margin integrity) of the tested adhesives in demineralized and not demineralized (sound) enamel after different selective enamel etching times are shown in Figure 2.

In demineralized enamel, iBond Self Etch attained a significantly lower percentage of continuous margins than in sound enamel when no selective enamel etching was performed (*p* = 0.003). Selective enamel etching significantly increased margin integrity of iBond Self Etch in both sound (*p* < 0.001) and demineralized enamel (*p* = < 0.001–0.003). Furthermore, a significant increase in margin integrity with increased etching times was observed for iBond Self Etch in demineralized enamel. Margin integrity of iBond Self Etch in demineralized enamel etched for 10 s was similar to that in non-etched sound enamel (*p* = 0.930). When demineralized enamel was etched for at least 30 s, margin integrity of iBond Self Etch surpassed that of non-etched sound enamel (*p* = < 0.001–0.008). With etching times of 60 and 120 s in demineralized enamel, iBond Self Etch attained similar margin integrity as in etched sound enamel (*p* = 0.839 and *p* = 0.372, respectively).

**Figure 3:**
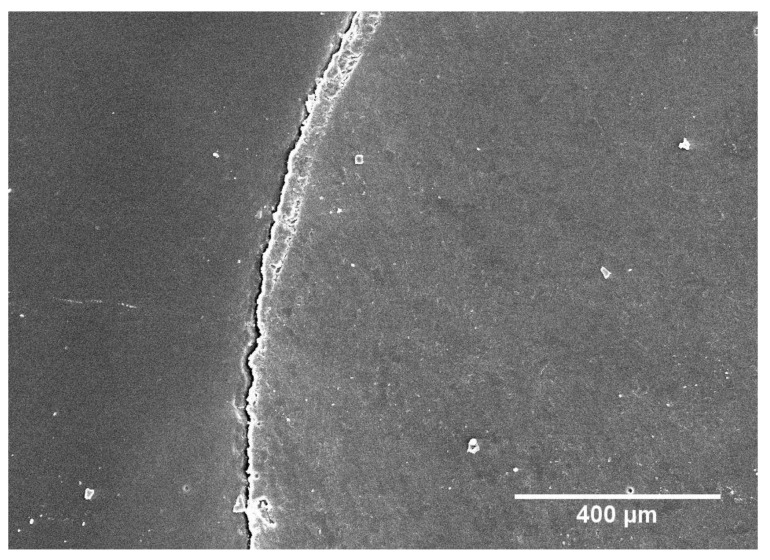
SEM micrograph of non-continuous margin (group 1; iBond Self Etch without phosphoric acid etching in demineralized enamel).

**Figure 4:**
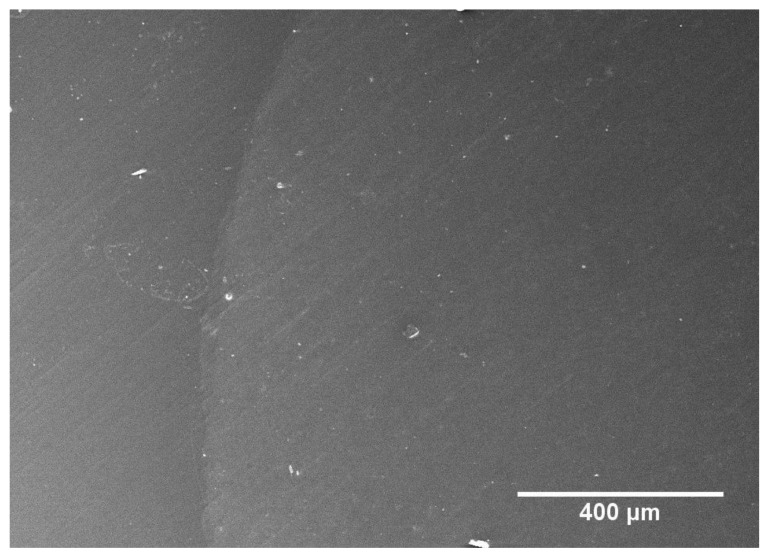
SEM micrograph of continuous margin (group 9; Clearfil SE Bond with 10 s phosphoric acid etching in demineralized enamel).

For Clearfil SE Bond, selective enamel etching also resulted in a significant increase in continuous margins in both sound (*p* < 0.001) and demineralized enamel (*p* < 0.001 each). However, in contrast to iBond Self Etch, the duration of enamel etching had no effect on margin integrity in demineralized enamel. All etched groups treated with Clearfil SE Bond showed significantly higher percentages of continuous margins in demineralized enamel than in non-etched sound enamel (*p* < 0.001 each), and similar margin integrity as in etched sound enamel (*p* = 0.59–1.00). Finally, comparing the adhesive systems iBond Self Etch and Clearfil SE Bond, the latter produced significantly higher percentages of continuous margins (*p* < 0.001 for all analogous groups in sound and demineralized enamel). Representative SEM micrographs of non-continuous and continuous margins are shown in Figure 3 and Figure 4, respectively.

## 4. Discussion

Various clinical approaches, including specific incremental placement techniques [24] and modulated light-curing procedures [25], have been suggested to control the development of polymerization-induced shrinkage stress in composite restorations in an attempt to prevent microleakage that has been associated with secondary caries [10]. Furthermore, in order to prevent bacterial colonization, it is important to improve the bonding of adhesives to dental hard tissues and optimize the margin integrity of restorations [26]. The present study demonstrated that enamel etching with phosphoric acid improves margin integrity of composite restorations in demineralized enamel when bonded with a one-step or two-step self-etch adhesive, respectively. Thus, the null hypothesis was rejected.

Crowns of bovine teeth were used in the current investigation. Bovine teeth are the most widely used substitute for human teeth in dental research and several studies showed that they are a suitable alternative [27]. Their large flat surfaces and the absence of caries were important advantages for creating standardized cavities and demineralized areas. Artificial enamel lesions as created in this study have been shown to possess a histological structure similar to enamel caries and white spot lesions [14,17,28,29]. In addition, the chosen thermocycling protocol is an established method to artificially age composite restorations and stress the interface to dental hard tissues [14,17,30]. Finally, the fabrication of replicas and subsequent margin analysis using scanning electron microscopy represents a precise and reliable non-destructive technique to assess margin integrity, as proven in numerous in vitro studies [19,20,21,31].

In accordance with a recent study [14], our findings show that margin integrity of composite restorations bonded with a one-step self-etch adhesive was significantly lower in demineralized enamel than in sound enamel. Pre-etching with phosphoric acid increased margin integrity of restorations bonded with the tested one- and two-step self-etch adhesives in both sound and demineralized enamel. Particularly interesting here is that by pre-etching demineralized enamel, similar percentages of continuous margins could often be achieved as in pre-etched sound enamel, and in most cases higher margin integrity was obtained than in non-pre-etched sound enamel. Thus, pre-etching enamel prior to the conventional protocol for the tested self-etch adhesives could improve the margin quality of composite restorations. In the reference groups with sound enamel, besides the non-etched groups, a standard 10 s selective enamel etching protocol before application of the adhesives was chosen, thereby following previous research [32,33].

It has been suggested, that the etching effect of self-etch adhesives is compromised by a demineralized surface layer, which may impede adequate penetration of the bonding agent [14]. Thus, acidic monomers incorporated in self-etch adhesives only produce a shallow and inhomogeneous etching pattern in enamel, leading to comparatively low micro-mechanical retention and limited bond durability [34,35]. On the other hand, phosphoric acid etching ensures deeper dissolution of enamel prism cores, which allows deeper penetration of the bonding agent and consequently improved microretention of the restoration [36,37]. This may explain the superior margin integrity of composite restorations after pre-etching both sound and demineralized enamel with phosphoric acid. A recent study found that the effect of enamel pre-etching with phosphoric acid on the bond strength of self-etch adhesives differs according to the primer pH [38]. In their study, the authors observed a significant increase in enamel bond strength after pre-etching enamel with phosphoric acid when ‘mild’ or ‘intermediately strong’ adhesives were subsequently applied, but not when a ‘strong’, i.e., highly acidic, adhesive was used [38]. The adhesives used in the present study, iBond Self Etch (pH = 1.6–1.8) and Clearfil SE Bond (pH = 2.0), which benefited substantially from enamel pre-etching, fall into the group of ‘intermediately strong’ and ‘mild’ adhesives, respectively [39]. The effect of enamel pre-etching with phosphoric acid on the margin integrity of composite restorations in demineralized enamel when bonded with more acidic (‘strong’) adhesives is, as of yet, unknown and should be investigated in subsequent studies.

Not only the fact whether or not phosphoric etching was performed in demineralized enamel, but also the etching duration affected margin integrity of the restorations bonded with the tested one-step self-etch adhesive, with a significant increase in margin quality being observed with increased etching times. A previous study compared the roughness of enamel surfaces etched with 37% phosphoric acid for different etching times and demonstrated that increased surface roughness was achieved with extended etching times up to 60 s [40]. Enhanced surface roughness after extended phosphoric acid times might increase enamel bond strength [36], which may have contributed to improved margin integrity of the composite restorations [41,42].

In the present study, the tested two-step self-etch adhesive resulted in significantly higher percentages of continuous margins than the investigated one-step self-etch adhesive. Previous studies showed that even after light curing, one-step self-etch adhesives were hydrophilic to the extent that water or dentin liquor could penetrate the adhesive layer, which could impair adhesion of restorations to tooth substance [43,44]. This problem might be avoided by using a two-step self-etch adhesive, since the application of a hydrophilic primer is followed by a hydrophobic bonding agent. Another reason for the superior margin integrity of specimens pretreated with the two-step self-etch adhesive Clearfil SE Bond may be that, unlike iBond Self Etch, it contains the functional monomer 10-methacryloyloxydecyl dihydrogen phosphate (10-MDP). 10-MDP monomers were shown to have a chemical structure capable of establishing particularly strong and stable chemical interactions with hydroxyapatite, which enables high and durable bond strength to dental hard tissues [45,46,47].

A limitation of the present study is the fact that it was performed in vitro. It is therefore important to verify the results obtained in this laboratory study in subsequent in vivo studies. Furthermore, only one representative of one-step and two-step self-etch adhesives was examined, respectively, which precludes generalizations of the results to other adhesives with different compositions. The fact that the present work for the first time systematically investigated the effect of phosphoric acid etching of demineralized enamel on the marginal integrity of composite restorations bonded with self-etch adhesives is a major strength of this study. Future studies should evaluate not only the margin integrity of composite restorations in sound and demineralized enamel, but also in demineralized enamel which underwent a remineralization procedure [48].

## 5. Conclusions

Based on the findings of this in vitro study, it can be concluded that enamel etching with phosphoric acid improves margin integrity of composite restorations in demineralized enamel when bonded with the examined one-step and two-step self-etch adhesives, respectively. Moreover, by pre-etching demineralized enamel, similar margin integrity can be achieved as in sound enamel.

## Figures and Tables

**Table 1:** Composition of the adhesives used in the present study as per manufacturers’ information.

Adhesive	Composition	pH	LOT No.	Manufacturer
iBond Self Etch	UDMA, 4-META, glutaraldehyde, acetone, water, photo-initiators, stabilizers	1.6–1.8	010902	Kulzer, Hanau, Germany
Clearfil SE Bond	Primer: 10-MDP, HEMA, hydrophilic aliphatic dimethacrylate, di-camphorquinone, DEPT, water	2	CB0279	Kuraray, Osaka, Japan
	Bonding: 10-MDP, Bis-GMA, HEMA, hydrophobic aliphatic dimethacrylate, di-camphorquinone, DEPT, colloidal silica		C50447	

UDMA: urethane dimethacrylate; 4-META: 4-methacryloyloxethyl trimellitate anhydride; 10-MDP: 10-methacryloyloxydecyl dihydrogen phosphate; HEMA: 2-hydroxyethyl methacrylate; DEPT: N,N-diethanol-p-toluidine; Bis-GMA: bisphenol-A-glycidyldimethacrylate.
